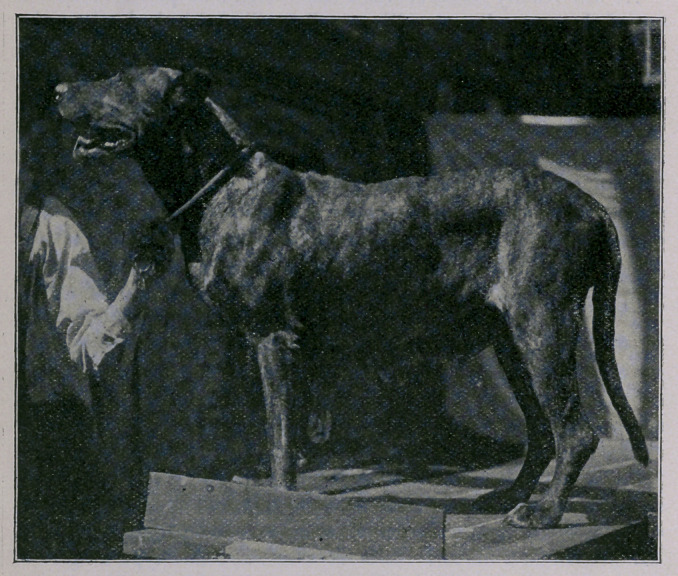# Department of Canine, Feline, and Avian Medicine and Surgery

**Published:** 1902-09

**Authors:** Cecil French

**Affiliations:** D.V.S. (McGill and Munich), Washington, D. C.


### DEPARTMENT OF CANINE, FELINE, AND AVIAN MEDICINE AND SURGERY.

By Cecil French, D.V.S. (McGill and Munich),
WASHINGTON, D. C.

Chronic Galactophoritis in the Bitch. The accompanying picture illustrates a condition I have met with recently which is nowhere described in text-books, though it is one which, on account of its unsightliness, calls for surgical interference.

As will readily be seen, it is a condition of neoplasia limited to one of the pectoral teats. We are all familiar with the hypertrophy of the teats in animals which have nursed numerous offspring, but in this instance the increase in volume of the teat is out of all proportion. Neoplastic growths are not at all uncommon in the mammary gland proper. They may be of innocent or malignant types, and there is every reason to believe that in some instances the former may undergo transformation into the latter. The innocent growths consist of fibroma, myxoma, lipoma, adenoma, or, very often, mixtures of these, and cysts. They are characterized by slow development, freedom from pain, and, after attainment of a certain size, quiescence. The malignant growths comprise sarcoma, carcinoma, and chondroma, the latter often exhibiting osseous elements. They are distinguishable from the former type by their more rapid growth, though they sometimes have periods of quiescence, by extension to neighboring lymphatics and by the pain and emaciation they induce, and by their tendency to ulcerate and undergo generalization.

The commonest of all these varieties is the fibroma, or, more correctly speaking, chronic interstitial fibrosis. Formerly much confusion existed as to its proper classification, and it was not infrequently confounded with adenofibroma; but, inasmuch as the essential element in a fibroma is fibrous tissue, and this type of growth develops as a pericanalicular fibrosis or proliferation and projection of connective tissue round the glandular acini, it is now recognized as pure fibroma. By this process of fibrosis groups of acini become isolated, and these, undergoing compression, lose their glandular structure and appear as "islands" of cells. It was these "islands" of cells which were once mistaken for true neoplastic or adenomatous formations.

In the case which forms the subject of this communication I removed the greatly elongated and enlarged teat and submitted the same to the McGill Pathological Laboratory for microscopic diagnosis. A report was rendered by Dr. A. G. Nicholls, of that institution. This showed the neoplasm in question to be identical with the type of growth so commonly met with in the gland proper—viz., a slowly progressive inflammatory fibrosis, or hyperplasia. The specimen showed some dilatation of the milk-ducts, into the cavities of which much desquamation of cells had taken place. In the connective tissue about the glandular elements was a very extensive extravasation of inflammatory leucocytes, which were very numerous near the ducts, and gradually diminishing in numbers as the distance increased from the centre.

There is a noteworthy point about this growth which would seem to indicate that it may be to a certain extent hereditary, for the animal from which it was taken is a grand-daughter of the stud-dog "Earl of Wurtemburg," her registered number being 66,162, while I have in my possession a bitch of the same breed, No. 69,200, sired by the same dog; and she also has a distinct hyperplasia of the very same teat, though not so pronounced. In other words, this strain of Great Danes may have a predisposition to develop this form of growth.

## Figures and Tables

**Figure f1:**